# Supplementary material for: Mid-Pregnancy Fructosamine Measurement—Predictive Value for Gestational Diabetes and Association with Postpartum Glycemic Indices
Source: Nutrients. 2018 Dec 18;10(12):2003. doi: 10.3390/nu10122003 (PMC6315870; doi:10.3390/nu10122003)
Supplement: Supplementary file 1 [file nutrients-10-02003-s001.pdf]

Supplementary Tables

Table S1. Unadjusted and adjusted associations of glucose exposures with fructosamine levels in mid-pregnancy, without vs. with IPW.

	**Without IPW**		**With IPW**	
	Unadjusted	Adjusted[1]	Unadjusted	Adjusted[1]
	β (95% CI)		β (95% CI)	
**Exposure**				
1h-glucose after GCT (per SD)	2.7 (0.3, 5.0)	3.4 (1.0, 5.8)	2.9 (0.5, 5.2)	3.6 (1.2, 6.0)
Abnormal GCT result (> vs. ≤ 140 mg/dl)	5.5 (−0.8, 11.7)	7.1 (0.7, 13.5)	5.8 (−0.4, 12.1)	7.5 (1.1, 13.9)
GDM (vs. IGT/IH/normal glycemia)	11.6 (0.9, 22.4)	14.7 (3.7, 25.6)	12.4 (1.8, 23.1)	15.5 (4.7, 26.3)

IPW = inverse probability weighting; [1]Adjusted for maternal age, race/ethnicity, parity, smoking during pregnancy, first trimester weight gain and maternal pre-pregnancy BMI.

Table S2. ROC curve (AUC) values for the predictive value of fructosamine levels, without vs. with IPW.

**Outcome**		**Without IPW**	**With IPW**
		ROC curve (AUC)	ROC curve (AUC)
GDM		0.52	0.52
	Stratified by GDM status		
Macrosomia	GDM = Yes	0.48	0.48
Macrosomia	GDM = No	0.52	0.52
LGA	GDM = Yes	0.59	0.59
LGA	GDM = No	0.53	0.53
SGA	GDM = Yes	0.57	0.57
SGA	GDM = No	0.51	0.51
Preterm (< 37 weeks)	GDM = Yes	0.46	0.46
Preterm (< 37 weeks)	GDM = No	0.50	0.50
Cesarean section	GDM = Yes	0.58	0.42
Cesarean section	GDM = No	0.54	0.54

IPW = inverse probability weighting; ROC = receiver operating characteristic; AUC = area under the ROC curve.

Table S3. Multivariable associations of fructosamine and GCT levels in mid-pregnancy with birth weight and birth weight for gestational age z score, without vs. with IPW.

**Exposure, per 1 SD**	**Outcome**	***Stratified by GDM status***	**Without IPW**	**With IPW**
			β (95% CI) [1]	β (95% CI) [1]
Fructosamine	Birthweight, g	GDM = No	5.7 (-22.5, 33.9)	6.8 (-21.4, 34.9)
Fructosamine	Birthweight, g	GDM = Yes	69.6 (-16.9,156.1)	64.5 (-20.4,149.5)
Fructosamine	BW for GA z-score	GDM = No	0.00 (-0.05, 0.05)	0.01 (-0.04, 0.06)
Fructosamine	BW for GA z-score	GDM = Yes	0.11 (-0.05, 0.27)	0.10 (-0.06, 0.25)
GCT	BW for GA z-score	GDM = No	54.2 (24.2, 84.1)	53.5 (23.5, 83.4)
GCT	BW for GA z-score	GDM = Yes	30.0 (-143,203.0)	20.9 (-151,192.3)
GCT	BW for GA z-score	GDM = No	0.11 (0.05, 0.16)	0.11 (0.05, 0.16)
GCT	BW for GA z-score	GDM = Yes	-0.09 (-0.40, 0.22)	-0.08 (-0.40, 0.23)

IPW = inverse probability weighting; GCT = glucose challenge test; [1] Adjusted for maternal age, race/ethnicity, parity, smoking during pregnancy, first trimester weight gain and maternal pre-pregnancy BMI.

Table S4. Multivariable associations of fructosamine and GCT levels in mid-pregnancy with maternal 3-year postpartum glycemic indices, without vs. with IPW (N = 451 [1]).

	Without IPW	With IPW	Without IPW	With IPW
	**Fructosamine, per 1 SD increment**		**GCT Result, per 1 SD increment**	
	β (95% CI) [2]		β (95% CI) [2]	
A1c, %	0.03 [0.00, 0.05]	0.02 (0.00, 0.05)	0.04 [0.02, 0.07]	0.05 (0.02, 0.07)
Fasting glucose, mg/dl	2.7 [-0.1, 5.5]	3.3 (0.4, 6.1)	3.6 [0.8, 6.4]	4.1 (1.2, 7.0)
	% difference (95% CI) [2]		% difference (95% CI) [2]	
Log fasting insulin	12.3 [2.1, 23.5]	13.4 (3.2, 24.7)	11.4 [1.1, 22.7]	12.5 (2.2, 23.9)
Log HOMA-IR	15.7 [3.7, 29.0]	17.7 (5.6, 31.3)	17.6 [5.4, 31.2]	19.7 (7.2, 33.5)

IPW = inverse probability weighting; GCT = glucose challenge test; [1] N = 132 for fasting glucose, fasting insulin and HOMA-IR and N = 450 for A1c; [2] Adjusted for maternal age, race/ethnicity, parity, smoking during pregnancy, first trimester weight gain and maternal pre-pregnancy BMI.
